# General-Purpose Bayesian Tensor Learning With Automatic Rank Determination and Uncertainty Quantification

**DOI:** 10.3389/frai.2021.668353

**Published:** 2022-01-07

**Authors:** Kaiqi Zhang, Cole Hawkins, Zheng Zhang

**Affiliations:** ^1^Department of Electrical and Computer Engineering, University of California, Santa Barbara, Santa Barbara, CA, United States; ^2^Department of Mathematics, University of California, Santa Barbara, Santa Barbara, CA, United States

**Keywords:** deep learning, tensor decomposition, tensor learning, Bayesian inference, uncertainty quantification

## Abstract

A major challenge in many machine learning tasks is that the model expressive power depends on model size. Low-rank tensor methods are an efficient tool for handling the curse of dimensionality in many large-scale machine learning models. The major challenges in training a tensor learning model include how to process the high-volume data, how to determine the tensor rank automatically, and how to estimate the uncertainty of the results. While existing tensor learning focuses on a specific task, this paper proposes a generic Bayesian framework that can be employed to solve a broad class of tensor learning problems such as tensor completion, tensor regression, and tensorized neural networks. We develop a low-rank tensor prior for automatic rank determination in nonlinear problems. Our method is implemented with both stochastic gradient Hamiltonian Monte Carlo (SGHMC) and Stein Variational Gradient Descent (SVGD). We compare the automatic rank determination and uncertainty quantification of these two solvers. We demonstrate that our proposed method can determine the tensor rank automatically and can quantify the uncertainty of the obtained results. We validate our framework on tensor completion tasks and tensorized neural network training tasks.

## 1. Introduction

Tensors (Kolda and Bader, 2009) are a generalization of matrices to describe and process multidimensional data arrays. Due to its ability to represent a huge amount of data by low-rank factorization, tensor computation has been applied in data recovery and compression (Acar et al., 2011; Jain and Oh, 2014; Austin et al., 2016), machine learning (Cichocki, 2014; Novikov et al., 2015; Sidiropoulos et al., 2017), uncertainty quantification (Zhang et al., 2014, 2016), and so forth. However, most of the existing tensor algorithms rely on numerical optimization, and estimating the tensor rank exactly is NP-Hard in some tensor formats (Hillar and Lim, 2013).

To overcome the rank determination challenge, Bayesian methods have been employed successfully in tensor completion tasks (Chu and Ghahramani, 2009; Xiong et al., 2010; Rai et al., 2014; Zhao et al., 2015a,c; Hawkins and Zhang, 2018; Gilbert and Wells, 2019). The key idea is to represent the tensor factors as some hidden statistical variables and to automatically determine the tensor ranks based on the training data and a proper rank-shrinking prior density. Chu and Ghahramani (2009) proposed a maximum a posteriori (MAP) MAP estimation, but a point prediction cannot estimate the uncertainty. In order to estimate model uncertainties, Gibbs sampling and mean-field approximate Bayesian methods have been employed in Xiong et al. (2010), Rai et al. (2014), and Zhao et al. (2015a,b), respectively. The former assumes that a conditional posterior density function can be obtained analytically and be sampled from easily. The latter assumes that the hidden parameters are mutually independent to each other. These methods work well in some simple tensor learning tasks such as tensor completion and factorization, but the may become over-simplified when solving more complicated problems such as tensorized neural networks.

**Paper Contributions**. This paper presents a Bayesian framework that is applicable to a broad class of tensor learning problems, e.g., tensor factorization/completion, tensor regression, and tensorized neural networks. Given a tensor learning model with a specified prior density, likelihood function and low-rank tensor approximation format, our framework estimates the posterior distribution of all the factors and automatically determine the tensor ranks via more flexible Bayesian inference methods such as Hamiltonian Monte Carlo (HMC) (Duane et al., 1987) and Stein Variational Gradient Descent (SVGD) (Liu and Wang, 2016). Compared with the mean-field Bayesian tensor completion (Zhao et al., 2015a,b), our tensor learning approach is more flexible because it does not require the strong assumption of independent hidden parameters. Further, our approach can be applied to highly non-linear tensor problems, i.e., tensorized neural networks. Due to the huge amount of training data in many tensor learning problems, estimating the full gradient can be computationally expensive. Therefore, we replace the full gradient in a tensor learning problem with the stochastic gradient (Chen et al., 2014) while achieving a similar level of accuracy. Because of that, our method has larger scalability than traditional Bayesian methods. Our HMC-based sampling approach to tensor learning returns a set of random samples following the posterior distribution, therefore, we can provide uncertainty estimations for both the model parameters and the predictive results. In certain situations HMC may require that the user store too many model copies for inference. For these situations we also develop an SVGD-based framework which applies deterministic updates to produce a small-sized set of particles to approximate the posterior distribution and approximate model uncertainty. We compare both approaches in our experiments. We note that sampling-based approaches have been employed in Bayesian neural networks (Neal, 1992; Liu and Wang, 2016) and data compression (Schmidt and Mohamed, 2009; Şimşekli et al., 2015). However, a thorough investigation of the generalized Bayesian tensor learning problem has not been reported. Our method have several advantages compared with existing methods: (1) **generic**–our method framework can deal with a broad class of tensor learning problems, including the tensor decomposition, tensor completion, tensor regression, tensorized neural networks, etc. (2) **scalable**–the stochastic-gradient implementation enables us to handle tensor learning problems with massive data. (3) **uncertainty-aware**–the resulting posterior samples provide uncertainty estimations for both the model parameters and predictive results.

This manuscript is an extended version of our recent work (Hawkins and Zhang, 2021), which reported SVGD training for Bayesian tensorized neural networks. Our manuscript extends Hawkins and Zhang (2021) in the following ways

1. In our previous work, we tested only one Bayesian sampler (SVGD). In this work, we compare two Bayesian samplers which have different memory/compute trade offs during both the training and inference stages of model deployment.
2. In our previous work, we considered only one tensor format (tensor-train) and one rank determination task (tensorized neural networks). In order to test the generality of our method we test our methods on both the tensor-train and Tucker formats and in two rank determination settings: tensor completion and tensorized neural networks.
3. In our previous work, the rank-threshold operation required a user-defined cutoff. We introduce a rank-thresholding cutoff that requires no user intervention.

## 2. Generalized Bayesian Tensor Learning

This section will present the model and numerical solver of our generic Bayesian tensor learning framework. We will demonstrate specific applications of our framework in later sections.

### 2.1. A Generalized Bayesian Model

We consider a generalized tensor learning problem: given a set of observed data D = {*D*_1_, *D*_2_, …*D*_*N*_}, we want to estimate the posterior density *p*(X|D) of an unknown tensor $X\in {\mathbb{R}}^{I_{1}\times I_{2}\times \cdots I_{d}}$. Because X has a huge number of unknown variables, directly solving the Bayesian tensor learning problem is computationally expensive.

Our framework allows various kinds of low-rank tensor representations (Kolda and Bader, 2009), such as CANDECOMP/PARAFAC (CP) (Harshman et al., 1970), tensor-train (TT) (Oseledets, 2011), and Tucker (Tucker, 1966). A low-rank tensor representation can significantly reduce the number of unknown variables. For instance, low-rank CP and tensor-train representations may reduce the number of unknowns from an exponential function of *d* to a linear one. In a general setting, we denote all model parameters (including the tensor factors and some additional hyper-parameters such as noise level or rank controlling parameters) as Θ, and the unknown tensor can be written as X(Θ). Then our goal is to estimate the posterior density

(1) $$\begin{array}{r} P\left(\Theta |D\right)\propto \overset{N}{\underset{n=1}{\prod }}P\left(D_{n}|\Theta \right)P\left(\Theta \right). \end{array}$$

Here $P\left(D|\Theta \right)=\overset{N}{\underset{n=1}{\prod }}P\left(D_{n}|\Theta \right)$ is a likelihood function, *P*(Θ) is a prior probability density. A key advantage of this Bayesian parameterized description is as follows: by properly choosing a prior density *P*(Θ), one can control the structure of Θ and thus automatically enforce a low-rank representation for X(Θ) based on the observed data D. Doing so overcomes the difficulty of rank determination in optimization-based tensor learning.

The formulation (1) is very generic. In practice, one only needs to specify the following information in order to use our Bayesian tensor learning framework:

- The learning task, such as tensor completion, multi-task tensor learning, tensorized neural networks for classification or regression, etc;
- A low-rank parameterization format, such as CP, Tucker, tensor-train factorization, etc;
- A prior density *P*(Θ) for tensor factors and hyper-parameters.

The first two decide the likelihood function *P*(D|Θ), and we will make it clear in section 3. The third decides how compact the resulting model would be: a stronger low-rank prior could result in a model with much fewer model parameters.

### 2.2. Stochastic Gradient HMC (SGHMC) Solver

Now we need to estimate the hidden tensor factors and hyper-parameters by computing the posterior density in (1). Existing methods (Zhao et al., 2015a; Hawkins and Zhang, 2018) do not apply to generalized tensor learning problems because they rely on Bayesian models that make strong assumptions about the posterior density and require linear models. The first Bayesian solver we employ is the Hamiltonian Monte Carlo (HMC) (Duane et al., 1987) to make our framework applicable to a broad class of tensor learning problems. HMC is an extension to Markov chain Monte Carlo (MCMC) Andrieu et al. (2003), and it uses the gradient information to increase efficiency.

**Algorithm 1:**
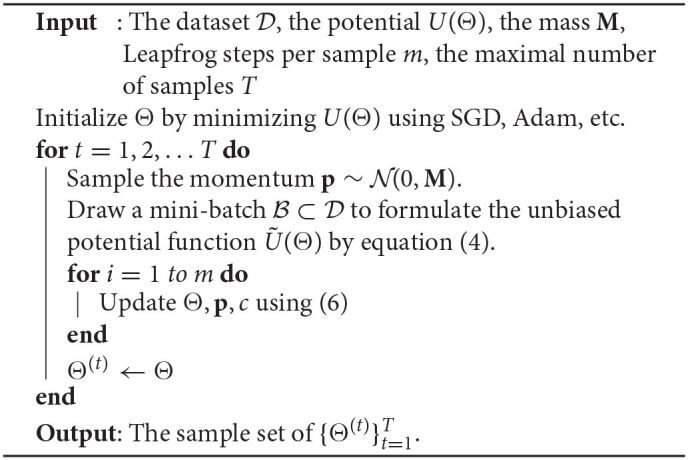
SGHMC with thermostats

The HMC method avoids the random walks in a standard MCMC framework by simulating the following dynamic system:

(2) $$\begin{array}{r} \frac{d\Theta }{dt}={\text{M}}^{-1}\text{p},\;\frac{d\text{p}}{dt}=-\nabla U\left(\Theta \right). \end{array}$$

Here **p** is the auxiliary momentum variable with the same dimension as Θ, **M** is a mass matrix. Here *U*(Θ) is the potential energy which is equal to the negative log posterior:

(3) $$\begin{array}{r} U\left(\Theta \right)=-\log P\left(\Theta |D\right)=-\overset{N}{\underset{n=1}{\sum }}\log P\left(D_{n}|\Theta \right)-\log P\left(\Theta \right). \end{array}$$

The HMC method starts from a random initial guess of Θ to simulate a sample of Θ, and its steady-state distribution converges to our desired posterior density *P*(Θ|D).

Standard HMC becomes inefficient when we solve a tensor learning problem with massive training samples, because computing the gradient requires estimating ∇ log *P*(*D*_*n*_|Θ) for every index *n* over the whole data set. This often happens in completing a huge-size tensor data set or training a tensorized neural network. To reduce the cost, we use the stochastic unbiased estimator of *U*(Θ):

(4) $$\begin{array}{r} Ũ\left(\Theta \right)=-\frac{N}{\left|B\right|}\underset{D_{i}\in B}{\sum }\log P\left(D_{i}|\Theta \right)-\log P\left(\Theta \right)+\text{const}. \end{array}$$

Here B ⊂ D denotes a mini-batch with |B| ≪ *N*. Then one can update the parameters *via* $\frac{d\Theta }{dt}={\text{M}}^{-1}\text{p}$ and $\frac{d\text{p}}{dt}=-\nabla Ũ\left(\Theta \right)$. To compensate the noise introduced by the stochastic gradient, we adopt the thermostats method (Ding et al., 2014) for our tensor learning framework. Specifically, a friction term *c* is introduced, i.e.,

(5) $$\begin{array}{l} \begin{array}{c} \frac{d\Theta }{dt}={\text{M}}^{-1}\text{p},\;\frac{d\text{p}}{dt}=-\nabla Ũ\left(\Theta \right)-c\text{p},\frac{dc}{dt}\\ \;=\frac{1}{\left|\Theta \right|}\text{tr}\left({\text{p}}^{T}{\text{M}}^{-1}\text{p}\right)-1. \end{array} \end{array}$$

The friction term changes accordingly to keep the average kinetic energy $\frac{1}{2}{\text{p}}^{T}{\text{M}}^{-1}\text{p}$ constant, thus keeping the distribution of samples invariant. Our framework employs a slightly modified leapfrog approach Iserles (1986) to solve the Hamiltonian system because it has a smaller integration error compared with other methods Duane et al. (1987):

(6) $$\begin{array}{l} \begin{array}{c} {\text{p}}_{t+\epsilon /2}\leftarrow {\text{p}}_{t}-\frac{1}{2}\epsilon \left(\nabla Ũ\left(\Theta_{t}\right)+c_{t}{\text{p}}_{t}\right),\;\Theta_{t+\epsilon }\leftarrow \Theta_{t}+\epsilon {\text{p}}_{t+\epsilon /2},\\ \;{\text{p}}_{t+\epsilon }\leftarrow {\text{p}}_{t+\epsilon /2}-\frac{1}{2}\epsilon \left(\nabla Ũ\left(\Theta_{t+\epsilon }\right)+c_{t}{\text{p}}_{t+\epsilon /2}\right),\\ \;c_{t+\epsilon }\leftarrow c_{t}+\epsilon \left(\frac{1}{\left|\Theta \right|}\text{tr}\left({\text{p}}^{T}{\text{M}}^{-1}\text{p}\right)-1\right), \end{array} \end{array}$$

where ϵ is the stepsize, and *t* is the iteration index.

### 2.3. Stein Variational Gradient Descent (SVGD) Solver

The HMC solver described in the previous section can accurately represent arbitrary distributions given a sufficient sampling budget. The disadvantage of the HMC approach is that it may require a large number of model copies for accurate uncertainty quantification (Neal, 1992). Therefore, we also consider the Stein Variational Gradient Descent (SVGD) (Liu and Wang, 2016) to approximate the posterior density *p*(**Θ**|D). SVGD uses a small number of particles to approximate a target distribution. The advantage of this approach compared to HMC is a lower memory cost due to a lower particle number. The disadvantage compared to HMC is a potential reduction in the accuracy of the final posterior representation.

**Algorithm 2:**
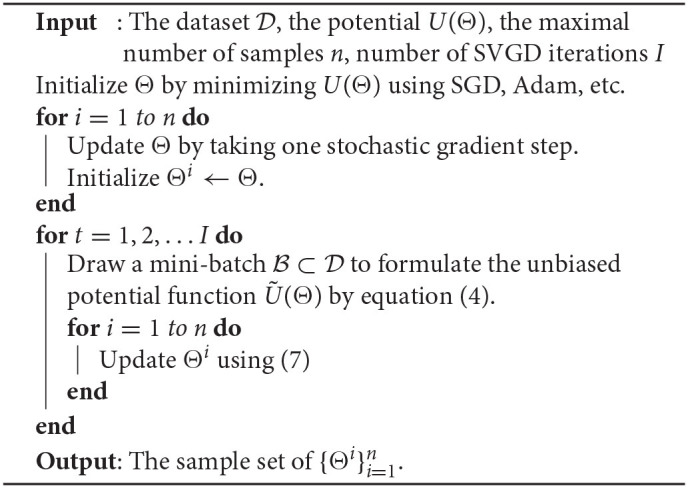
SVGD

SVGD aims to find a set of particles ${\left\{\Theta^{i}\right\}}_{i=1}^{n}$ such that $q\left(\Theta \right)=\frac{1}{n}\overset{n}{\underset{i=1}{\sum }}k\left(\Theta ,\Theta^{i}\right)$ approximates the true posterior *p*(**Θ**|D). Here *k*(·, ·) is a positive definite kernel, and we use the radial basis function kernel in this work. The particles can be found by minimizing the KL divergence between *q*(**Θ**) and *p*(**Θ**|D)). The optimal update ϕ(·) is derived in (Liu and Wang, 2016) and takes the form

(7) $$\begin{array}{l} \begin{array}{c} \Theta^{k}\leftarrow \Theta^{k}+\epsilon \phi \left(\Theta^{k}\right),\;\phi \left(\Theta^{k}\right)\\ \;=\frac{1}{n}\overset{n}{\underset{i=1}{\sum }}\left[k\left(\Theta^{i},\Theta^{k}\right)\nabla_{\Theta^{i}}U\left(\Theta^{i}\right)+\nabla_{\Theta^{i}}k\left(\Theta^{i},\Theta^{k}\right)\right] \end{array} \end{array}$$

where ϵ is the step size. The gradient of the potential function $\nabla_{\Theta^{i}}U\left(\Theta^{i}\right)$ can be approximated with a stochastic gradient $\nabla_{\Theta^{i}}Û\left(\Theta^{i}\right)$ when the data size |D| is large. The SVGD training update is, therefore, a deterministic function of the existing particle locations and minibatch gradients. The particle locations are incorporated through the kernel function *k*(·, ·) and the minibatch gradients of each individual particles are $\nabla_{\Theta^{i}}U\left(\Theta^{i}\right)$. To sample from the SVGD distribution during training or inference one computes a deterministic forward pass for each of the *n* particles ${\left\{\Theta \right\}}_{i=1}^{n}$. At each step of the SVGD Bayesian learning process we require a deterministic forward/backward pass for each particle to compute the gradient in Equation (7). In practice we use ten particles to compute our posterior approximation. Unlike the HMC update, in which we re-sample the momentum parameter, no additional noise is introduced during the training process.

We make two observations about the computational requirements of SVGD. Due to the update in Equation (7) this method requires the computation of *n* gradients of the potential energy function *U* at each step. Second, each computation requires that we acesss *n* particles. Therefore, the per-step memory and compute costs of the SVGD solver are *n*× as large as the per-step costs of the HMC solver. The advantage of the SVGD model is the compact final representation for uncertainty quantification (Liu and Wang, 2016).

## 3. Specifying Potential Energy Functions

One can run our Bayesian tensor learning framework as a black-box after specifying the energy function based on three components: a learning task, a low-rank tensor representation format, and a prior density. In this section, we will first give the details of two cases: Bayesian tensor completion with a Gaussian likelihood and low-rank CP representation, and Bayesian tensorized neural network classification with a multinomial likelihood and a low-rank tensor-train representation. Then we will also briefly show the Bayesian models for some other cases.

### 3.1. Bayesian CP Tensor Completion

Given D = {Ω, Y_Ω_} where Y_Ω_ denotes some partially observed noisy tensor elements and Ω specifies the sample indices, we aim to find a low-rank tensor X such that Y = X + E, where E denotes a Gaussian noise tensor with zero mean and variance σ. We use the CP factorization (Harshman et al., 1970; Bro, 1997) to paramterize X:

(8) $$\begin{array}{r} X=\overset{R}{\underset{r=1}{\sum }}{\text{a}}_{r}^{\left(1\right)}◦\cdots ◦{\text{a}}_{r}^{\left(d\right)}=⟦{\text{A}}^{\left(1\right)},\ldots ,{\text{A}}^{\left(d\right)}⟧, \end{array}$$

where ◦ denotes the outer product of vectors and ⟦·⟧ denotes a Kruskal operator. In the Bayesian tensor learning, we need to estimate CP factors ${\left\{{\text{A}}^{\left(k\right)}\right\}}_{k=1}^{d}$ and CP rank *R*. Following the Bayesian CP tensor completion (Zhao et al., 2015c), we set the hidden parameters as Θ = {**A**^(1)^, …, **A**^(*d*)^, Λ, τ}, where hyper-parameters Λ = diag(λ_1_, …, λ_*R*_) and τ = 1/σ control the tensor ranks and noise level, respectively. The Gaussian noise assumption leads to the Gaussian likelihood

(9) $$\begin{array}{r} \begin{array}{c} P\left(Y_{\Omega }|\Theta \right)=N\left(Y_{\Omega }|⟦{\text{A}}^{\left(1\right)},\ldots {\text{A}}^{\left(d\right)}⟧,\tau^{-1}\right) \end{array} \end{array}$$

where Ω denotes the observed tensor entries. The negative log likelihood associated with each observation is

(10) $$\begin{array}{r} -\log P\left(Y_{i}|\Theta \right)=\frac{1}{2}(\left(Y_{i}-f\left(i;\Theta \right){)}^{2}\tau -\log\tau \right)+\text{const}, \end{array}$$

where *f* denotes the forward evaluation from CP factors to the *i*-th element of tensor X.

Next, our goal is to develop a rank-shrinkage prior. The prior density will enforce structured sparsity on the CP factor matrices, leading to rank shrinkage. We define this rank-shrinkage prior density as

(11) $$\begin{array}{l} P\left(\Theta \right)=\prod_{k=1}^{d}\prod_{i_{k}=1}^{I_{k}}N\left({\text{A}}^{\left(k\right)}\left(i_{k},:\right)|0,\Lambda^{-1}\right)\\ \;\prod_{r=1}^{R}\text{Gamma}\left(\lambda_{r}|a,b\right)\text{Gamma}\left(\tau |c,d\right), \end{array}$$

We remark that the Gaussian prior on the factor matrices enforces that all factor matrix entries in the same column (same rank) share the same variance. Therefore, as the hyperparameter λ_*j*_ → ∞ all entries in the columns {**A**^(*k*)^(:, *j*)} shrink to 0.

This rank-shrinkage prior leads to the following negative log-priors:

(12) $$\begin{array}{l} \begin{array}{c} -\log P\left({\text{A}}^{\left(k\right)}|\Lambda \right)=\frac{1}{2}\overset{R}{\underset{r=1}{\sum }}\left(|{\text{A}}^{\left(k\right)}\left(:,r\right){|}^{2}\lambda_{r}-\overset{d}{\underset{k=1}{\sum }}I_{k}\log\lambda_{r}\right),\\ \;-\log P\left(\Lambda \right)=\overset{R}{\underset{r=1}{\sum }}-\left(a-1\right)\log\lambda_{r}+b\lambda_{r}. \end{array} \end{array}$$

The noise level τ may vary among different datasets, and λ_*r*_ can be very large for diminishing ranks. Therefore, we slightly modify the model to ensure the numerical stability in HMC-based Bayesian tensor learning. Instead of sampling τ and λ_*r*_ directly, we sample $\hat{\tau }=\log\left(\tau \right)$ and ${\hat{\lambda }}_{r}=\lambda_{r}^{-1}$ for better numerical stability, because these values can vary dramatically among different datasets. Therefore, we use the following prior distributions:

(13) $$\begin{array}{r} P\left(\hat{\tau }\right)=\frac{\exp\left(c\hat{\tau }\right)\exp\left(-e^{\hat{\tau }}\right)/d)}{d^{c}T\left(c\right)},P\left(\hat{\lambda }\right)=\frac{b^{a}}{T\left(a\right)}{\left(1/\hat{\lambda }\right)}^{a+1}\exp\left(-b/\hat{\lambda }\right). \end{array}$$

Combining equations (12) and (13), we have the modified negative log-prior

(14) $$\begin{array}{l} \begin{array}{c} -\log P\left(\Theta \right)=\overset{R}{\underset{r=1}{\sum }}(\frac{1}{2}\overset{d}{\underset{k=1}{\sum }}\left(|{\text{A}}^{\left(k\right)}\left(:,r\right){|}^{2}/{\hat{\lambda }}_{r}+I_{k}\log{\hat{\lambda }}_{r}\right)\\ \;+\left(\alpha +1\right)\log{\hat{\lambda }}_{r}+\frac{\beta }{{\hat{\lambda }}_{r}})\\ \;-c\hat{\tau }+\exp\hat{\tau }/d. \end{array} \end{array}$$

Based on the above prior density and our given Gaussian noise model, the potential function (neglecting the constants) for the Bayesian CP tensor completion is

(15) $$\begin{array}{l} \begin{array}{c} U\left(\Theta \right)=-\log P\left(\Theta |Y_{\Omega }\right)=-\log P\left(\Theta \right)-\underset{i\in \Omega }{\sum }\log P\left(Y_{i}|\Theta \right)\\ \;=\frac{1}{2}\left(||{\left(Y-⟦{\text{A}}^{\left(1\right)},\ldots ,{\text{A}}^{\left(d\right)}⟧\right)}_{\Omega }|{|}_{\text{F}}^{2}\exp\hat{\tau }-|\Omega |\hat{\tau }\right)\\ \;+\overset{R}{\underset{r=1}{\sum }}(\frac{1}{2}\overset{d}{\underset{k=1}{\sum }}\left(|{\text{A}}^{\left(k\right)}\left(:,r\right){|}^{2}/{\hat{\lambda }}_{r}+I_{k}\log{\hat{\lambda }}_{r}\right)\\ \;+\left(a+1\right)\log{\hat{\lambda }}_{r}+\frac{b}{{\hat{\lambda }}_{r}})-c\hat{\tau }+\exp\hat{\tau }/d. \end{array} \end{array}$$

We note that in order to use other low-rank tensor formats, all that is necessary is a change in the prior density. We provide the specific prior densities for the tensor-train and Tucker formats in later sections.

### 3.2. Bayesian Tensorized Neural Networks

We further show how to apply our Bayesian tensor learning to train a tensorized deep neural network in the tensor-train (TT) format. Given the training data $D={\left\{{\text{x}}_{n},{\text{y}}_{n}\right\}}_{n=1}^{N}$, we want to find a low-rank tensor W in the TT format to describe the weight matrices or convolution filters such that **y** = *g*(**x**, W), where *g* denotes the forward propagation model of a neural network.

For a weight matrix **W** of size *M* × *J*, one can decompose $M=\prod_{k=1}^{K}m_{k}$ and $J=\prod_{k=1}^{K}j_{k}$, then reformulate **W** as a 2*K*-dimension tensor W with size *m*_1_ × *j*_1_ × ⋯ × *m*_*K*_ × *j*_*K*_. Afterwards, W is approximated by a low-rank tensor-train decomposition

(16) $$\begin{array}{r} W=⟦G^{\left(1\right)},\ldots ,G^{\left(K\right)}{⟧}_{TT}\Leftrightarrow W\left(i_{1},\cdots \;,i_{K},l_{1},\cdots \;,l_{K}\right)\\ \;=G^{\left(1\right)}\left(:,i_{1},l_{1},:\right)\cdots G^{\left(K\right)}\left(:,i_{K},l_{K},:\right) \end{array}$$

where $G^{\left(k\right)}\in {\mathbb{R}}^{R_{k-1}\times m_{k}\times j_{k}\times R_{k}}$ is called the TT core, *R*_*k*_ is the TT rank, *R*_0_ = *R*_*K*_ = 1, and ⟦·⟧_*TT*_ denotes the tensor-train product (Oseledets, 2011). The convolutional layers can be decomposed in a similar way. The convolution kernel C is a 4-th dimension tensor in *M* × *J* × *H* × *W*, where *H* and *W* denote the height and width of the convolution window. This tensor can be further viewed as a (2*K* + 2)-dimensional tensor with size *m*_1_ × *j*_1_ × ⋯ × *m*_*K*_ × *j*_*K*_ × *H* × *W*. In our experiments *H* = *W* = 3 remain unchanged, and we only compress along the remaining dimensions, i.e.,

(17) $$\begin{array}{r} C=⟦G^{\left(1\right)},G^{\left(2\right)},\ldots ,G^{\left(2K\right)}{⟧}_{TT}. \end{array}$$

The shape of each factors G^(1)^, G^(2)^, …G^(2*K*−1)^, G^(2*K*)^ are *m*_1_ × *R*_1_, *R*_1_ × *j*_1_ × *R*_2_, …, *R*_2*K*−2_ × *m*_*K*_ × *R*_2*K*−1_, *R*_2*K*−1_ × *j*_*K*_ × *H* × *W*, respectively. The parameters in both fully connected layers and convolutional layers can be represented as

(18) $$\begin{array}{r} \Theta =\left\{G^{\left(1\right)},\Lambda^{\left(1\right)},G^{\left(2\right)},\Lambda^{\left(2\right)},\ldots \right\}, \end{array}$$

where hyper-parameters $\Lambda^{\left(k\right)}=\text{diag}\left(\lambda_{1}^{\left(k\right)},\ldots \lambda_{R_{k}}^{\left(k\right)}\right)$ are used to control the rank *R*_*k*_.

Here a Gaussian prior is placed over each tensor factor and a Gamma prior is placed over Λ^(*k*)^,

(19) $$\begin{array}{l} \begin{array}{c} P\left(G^{\left(k\right)}|\Lambda^{\left(k-1\right)},\Lambda^{\left(k\right)}\right)=\underset{i,j}{\prod }N(G^{\left(k\right)}\left(i,:,j\right)|0,{\left(c_{k}\lambda_{i}^{\left(k-1\right)}\lambda_{j}^{\left(k\right)}\right)}^{-1}),\\ \;P\left(\Lambda^{\left(k\right)}\right)=\overset{R_{k}}{\underset{r=1}{\prod }}\text{Gamma}\left(\lambda_{r}^{\left(k\right)}|\alpha ,\beta \right),\\ \;P\left(\Theta \right)=\overset{d}{\underset{k=1}{\prod }}P\left(G^{\left(k\right)}|\Lambda^{\left(k-1\right)},\Lambda^{\left(k\right)}\right)\overset{d-1}{\underset{k=1}{\prod }}P\left(\Lambda^{\left(k\right)}\right). \end{array} \end{array}$$

where α and β are constants. Once the estimated parameter $\lambda_{r}^{\left(k\right)}$ is larger than a threshold, we delete one horizontal slice of G^(*k*)^ and one frontal slice of G^(*k*+1)^. The distribution of Λ^(*k*)^ is the same as Equation (30). The prior of unknown parameters Θ = {G^(*k*)^, Λ^(*k*)^} is $P\left(\Theta \right)=\prod_{k=1}^{d}P\left(U^{\left(k\right)}|\Lambda^{\left(k\right)}\right)P\left(\Lambda^{\left(k\right)}\right)$. The idea of the tensor-train low-rank prior is structurally similar to the idea of the low-rank CP prior in Equation (12). However, instead of shrinking columns of every factor matrices, each element $\lambda_{j}^{\left(k\right)}$ controls the prior variance of two factor tensor slices, each of which can shrink to 0. The negative log prior is

(20) $$\begin{array}{l} \begin{array}{c} -\log P\left(\Theta \right)=\frac{1}{2}\overset{d}{\underset{k=1}{\sum }}\overset{I_{k}}{\underset{i=1}{\sum }}\langle G^{\left(k\right)}\left(:,i,:\right)*G^{\left(k\right)}\left(:,i,:\right),{\left(\Lambda^{\left(k-1\right)}⊗\Lambda^{\left(k\right)}\right)}^{-1}\rangle \\ \;-\frac{1}{2}\overset{d}{\underset{k=1}{\sum }}\left(I_{k}R_{k}\overset{R_{k-1}}{\underset{r=1}{\sum }}\lambda_{r}^{\left(k-1\right)}+I_{k}R_{k-1}\overset{R_{k}}{\underset{r=1}{\sum }}\lambda_{r}^{\left(k\right)}\right)\\ \;-\overset{d-1}{\underset{k=1}{\sum }}\overset{R_{k}}{\underset{r=1}{\sum }}\left(\left(a-1\right)\log\lambda_{r}^{\left(k\right)}-b\lambda_{r}^{\left(k\right)}\right). \end{array} \end{array}$$

Here ∗ denotes the element-wise product of two tensors or matrices.

For all hyperparameters $\lambda_{r}^{\left(k\right)}$, we sample ${\hat{\lambda }}_{r}^{\left(k\right)}=\log\lambda_{r}^{\left(k\right)}$ and use the log Gamma distribution as a prior. The potential function can be computed as

(21) $$\begin{array}{r} \begin{array}{c} U\left(\Theta \right)=-\log P\left(\Theta |D\right)=\overset{N}{\underset{n=1}{\sum }}\text{loss}\left({\text{y}}_{n},g\left({\text{x}}_{n},\Theta \right)\right)-\log P\left(\Theta \right), \end{array} \end{array}$$

where loss(·) is the negative log likelihood and *g*(·) denotes the neural network. The loss function can be the cross entropy loss for classification problems and the mean square error loss for regression problems. After getting the potential function, we can apply the SGHMC or SVGD framework to draw samples for the parameters Θ.

In this framework, the tensor ranks can be adjusted automatically to reduce the neural network model size in training. We propose to set a threshold and reduce the rank when

(22) $$\begin{array}{r} {\hat{\lambda }}_{r}^{\left(k\right)}\geq \log\left(\frac{1}{2}S_{k}R_{k-1}+\frac{1}{2}S_{k+1}R_{k+1}+\alpha \right)+\log\beta -\epsilon , \end{array}$$

where *S*_*k*_ = *M*_*k*_*J*_*k*_ for the fully connected layers and *S*_2*k*−1_ = *M*_*k*_, *S*_2*k*_ = *J*_*k*_ for the convolutional layers. We select this threshold for λ by setting G^*k*^(:, *i*, :) = 0 and maximizing the negative log prior from (20) with respect to Λ. Therefore, the threshold is chosen as an MAP point of the marginal log-prior conditioned on the value of the tensor factors.

### 3.3. More General Models

Our Bayesian tensor learning framework can also be applied to other low-rank tensorized neural network formats such as the CP and Tucker formats, other tensorized neural network tasks such as regression instead of classification, and to other tensor completion and factorization approaches using the tensor-train or Tucker formats. For instance, we only need to change the likelihood function to a Gaussian distribution when solving a neural network regression task. We summarize these results in Table 1. In this subsection we provide the likelihoods for more general tensor models and a Bayesian rank-shrinkage prior for the low-rank Tucker format.

**Table 1:** Equations to calculate the potential energy *U*(Θ) for different tensor learning tasks and with different low-rank tensor formats.

**Learning tasks**	**Likelihood**	**CP**	**Tucker**	**Tensor-Train**
Tensor completion	Gaussian	(12) + (14) +(10)	(31) + (14) + (10)	(20) + (14) + (10)
Neural network classification	Multinomial	(12) + (24)	(31) + (24)	(20) + (24)
Neural network regression	Gaussian	(12) + (26)	(31) + (26)	(20) + (26)

**Classification Problems**. In most classification problems, the neural network can give a likelihood $\hat{{\text{y}}_{i}}=f\left({\text{x}}_{i};\Theta \right)$ directly, where *f*(**x**_*i*_; Θ) is the propagation function of the network, ${\hat{\text{y}}}_{i}$ is a vector and each element denotes the probability that *x*_*i*_ belongs to one class. It is usually the softmax of output of the last linear layer. Suppose **y**_*i*_ is a vector with size *C*, *C* is the total number of classes,

(23) $$\begin{array}{l} {\text{y}}_{ic}=\{\begin{array}{l} 1,\text{if }{\text{x}}_{i}\text{ in class }c\\ 0,\text{ otherwise}. \end{array} \end{array}$$

The negative log likelihood is

(24) $$\begin{array}{r} -\log P\left({\text{y}}_{i}|\Theta \right)=\langle {\text{y}}_{i},-\log f\left(x_{i};\Theta \right)\rangle . \end{array}$$

**Regression Problems**. In a regression problem, it is usually assumed to have a Gaussian likelihood function

(25) $$\begin{array}{r} P\left({\text{y}}_{i}|\Theta \right)=N\left({\text{y}}_{i}|f\left({\text{x}}_{i};\Theta \right),\sigma^{2}\right). \end{array}$$

This leads to the following negative log-likelihood:

(26) $$\begin{array}{r} -\log P\left({\text{y}}_{i}|\Theta \right)=\frac{1}{2}{\left({\text{y}}_{i}-f\left({\text{x}}_{i};\Theta \right)\right)}^{2}/\sigma^{2}, \end{array}$$

where σ is a hyperparameter denoting the variance.

**Tucker Tensor Prior**. A popular alternative to the CP and tensor-train tensor formats is the low-rank Tucker format (Tucker, 1966). The Tucker decomposition projects the original tensor X into a smaller kernel tensor G,

(27) $$\begin{array}{l} \begin{array}{c} X=G\overset{d}{\underset{k=1}{⊗}}{\text{U}}^{\left(k\right)}\Leftrightarrow X\left(i_{1},\cdots \;,i_{d}\right)\\ \;=\overset{R_{1}}{\underset{r_{1}=1}{\sum }}\cdots \overset{R_{d}}{\underset{r_{d}=1}{\sum }}G\left(i_{1},\cdots \;,i_{d}\right){\text{U}}^{\left(1\right)}\left(i_{1},r_{1}\right)\cdots {\text{U}}^{\left(d\right)}\left(i_{d},r_{d}\right). \end{array} \end{array}$$

Similar to Zhao et al. (2015b), the priors of **U**^(*k*)^ and G are set as

(28) $$\begin{array}{r} P\left({\text{U}}^{\left(k\right)}|\Lambda^{\left(k\right)}\right)=\overset{I_{k}}{\underset{i_{k}=1}{\prod }}N\left({\text{U}}^{\left(k\right)}\left(i_{k},:\right)|0,{\left(\Lambda^{\left(k\right)}\right)}^{-1}\right) \end{array}$$

and

(29) $$\begin{array}{r} \begin{array}{c} P\left(G|\Lambda^{\left(1\right)},\ldots ,\Lambda^{\left(d\right)}\right)=\underset{r_{1},\ldots ,r_{d}}{\prod }N\left(G\left(r_{1},\ldots ,r_{d}\right)|0,\beta \overset{d}{\underset{k=1}{\prod }}{\left(\lambda_{r_{k}}^{\left(k\right)}\right)}^{-1}\right) \end{array} \end{array}$$

respectively. Here β is a constant scaling factor. For simplicity, we assume β is a constant instead of a random variable, which differs from Zhao et al. (2015b). The hyperparameter Λ^(*k*)^ follows from the Gamma distribution

(30) $$\begin{array}{r} \begin{array}{c} P\left(\Lambda^{\left(k\right)}\right)=\overset{R_{k}}{\underset{r=1}{\prod }}\text{Gamma}\left(\lambda_{r}^{\left(k\right)}|a,b\right). \end{array} \end{array}$$

Here, Λ^(*k*)^ is shared between **U**^(*k*)^ and G. We observe that similar to other low-rank tensor formats this prior enforces structural rank-shrinkage. This approach differs from the CP format in that each low-rank hyperparamter $\lambda_{r_{k}}^{\left(k\right)}$ controls entries in both a factor matrix and the Tucker tensor core G.

In summary, the prior of the unknown parameters Θ = {G, **U**^(*k*)^, Λ^(*k*)^} is

$$P\left(\Theta \right)=P\left(G|\Lambda^{\left(1\right)}\ldots \Lambda^{\left(1\right)}\right)\overset{d}{\underset{k=1}{\prod }}P\left({\text{U}}^{\left(k\right)}|\Lambda^{\left(k\right)}\right)P\left(\Lambda^{\left(k\right)}\right)$$

The negative log prior is

(31) $$\begin{array}{l} \begin{array}{c} -\log P\left(\Theta \right)=\frac{1}{2}\overset{d}{\underset{k=1}{\sum }}\text{tr}\left({\text{U}}^{\left(k\right)}\Lambda^{\left(k\right)}{\left({\text{U}}^{\left(k\right)}\right)}^{T}\right)+\frac{1}{2}\langle \beta \overset{d}{\underset{k=1}{⊗}}\Lambda^{\left(k\right)},G*G\rangle \\ \;-\frac{1}{2}\overset{d}{\underset{k=1}{\sum }}\left(\left(I_{k}+\underset{t\neq k}{\prod }R_{t}\right)\overset{R_{k}}{\underset{r=1}{\sum }}\log\lambda_{r}^{\left(k\right)}\right)\\ \;-\overset{d}{\underset{k=1}{\sum }}\overset{R_{k}}{\underset{r=1}{\sum }}\left(\left(a-1\right)\log\lambda_{r}^{\left(k\right)}-b\lambda_{r}^{\left(k\right)}\right) \end{array} \end{array}$$

where G ∗ G is the element-wise product of two tensors.

## 4. Numerical Experiments

### 4.1. Tensor Completion

We first consider a synthetic example and an MRI data experiment to show the efficiency of our proposed methods in tensor data recovery, uncertainty estimation, and automatic rank determination in CP tensor format. In both our SGHMC and SVGD implementations, we generate the initial guess by two steps: we first use the ADAM method (Kingma and Ba, 2014) to reach the neighborhood of a local optimal, and then reduce its rank by truncating all ${\hat{\lambda }}_{r}$ below the threshold given in Equation (22). After that, with SGHMC algorithm, we discard the first 50 samples and use the next 450 samples for evaluation. With SVGD algorithm, we use 10 samples for evaluation.

#### 4.1.1. Synthetic Dataset

We first randomly generated a 20 × 20 × 20 tensor with the ground truth rank of 5. We consider two sets of experiments. **Case 1: tensor factors with uniform distributions**. Assume the factors follow an independent uniform distribution between 0 and 1. We randomly select 10% of the tensor data to be observed. **Case 2: tensor factors with Gaussian distribution**. Assume the factors follow a Gaussian distribution with zero mean and variance one. We randomly sample 20% entries of the whole tensor. We select a higher observation ratio in this task because it is more difficult than task with uniformly distributed tensor factors. This is because the true data and the noise have similar distributions. In both cases, we perturb all elements with identically independent distributed Gaussian noise N(0, σ^2^). In Equation (11), we set *R* = 15, *a* = *c* = 1, *b* = 1 for the uniform factors and *b* = 4 for Gaussian factors, *d* = 10^6^ when σ = 0.001 and *d* = 10^4^ otherwise. For the HMC approach we divided the parameters in two groups, and set the mass of factors matrices as 1 and the mass of all other parameters to 100, as the values of the factor matrices vary more during sampling. We report the root mean square error

$$\text{RMSE}:||\bar{X}-Y|{|}_{F}/\sqrt{\underset{k}{\prod }I_{k}}$$

where Y is the ground truth and ||·||_*F*_ is the Frobenius norm, and SD is the predicted noise standard deviation. The results are shown in Table 2.

**Table 2:** Numerical results of tensor completion for the synthetic experiment and MRI dataset.

		**Proposed-HMC**			**Proposed-SVGD**			**BFCP (Zhao et al.**, **2015a****)**		
**True factors**	**Noise**	**Est. Rank**	**RMSE**	**SD**	**Est. Rank**	**RMSE**	**SD**	**Est. Rank**	**RMSE**	**SD**
	0.001	5	0.0013	0.0047	5	0.0019	0.0011	1	0.1476	0.1517
Uniform	0.003	5	0.0038	0.0040	5	0.0031	0.0016	1	0.1499	0.1607
Rank-5	0.01	5	0.0128	0.0118	5	0.0114	0.0098	1	0.1386	0.1365
	0.03	5	0.0403	0.0318	5	0.0512	0.0071	1	0.1468	0.1523
	0.001	5	0.0013	0.0025	5	0.0019	0.0007	6	0.0005	0.0011
Gaussian	0.003	5	0.0038	0.0031	5	0.0027	0.0019	6	0.0033	0.0033
Rank-5	0.01	5	0.0130	0.0102	5	0.0119	0.0069	5	0.0106	0.0110
	0.03	5	0.0418	0.0236	5	0.0336	0.0193	7	0.0338	0.0354
MRI dataset		65	0.0856	0.0670	65	0.0727	0.0319	17	0.1495	0.1456

For the tensor with Gaussian factors, our proposed methods perform almost as well as the mean field approximation (BFCP) (Zhao et al., 2015a) in terms of RMSE and SD. For the tensor with uniform-distributed factors, the BFCP method always underestimates the rank and results in high recovery error and SD. This is because the mean-field assumption on the posterior in the BFCP method places a strong Gaussian assumption on the approximating distribution. We also observe in Table 2 that the SVGD approach can produce lower RMSE estimates than the proposed HMC approach, but may underestimate the uncertainty and predict an SD that is too low.

#### 4.1.2. MRI Dataset

We continue to consider the PINCAT MRI dataset (Candes et al., 2013). This is a 128 × 128 × 50 complex-value tensor and we only consider its amplitude. We re-scale the tensor such that the average amplitude $||A|{|}_{F}/\sqrt{I_{1}I_{2}I_{3}}=1$. We randomly sample 80000 (≈ 10%) elements. The parameters in (11) are set to be *a* = 1, *b* = 0.2, *c* = 1, *d* = 10^4^, and *R* = 80. We compare our results with BFCP report them in Table 2. The results in Table 2 demonstrate that our methods obtain a much lower RMSE and SD than BFCP, and that BFCP underestimates the rank.

### 4.2. Tensorized Neural Networks

In this section, we present numerical experiments of our Bayesian tensor learning framework for tensorized neural network tasks. We evaluate both the compression capabilities and accuracy of our proposed method on two common computer vision datasets.

#### 4.2.1. Datasets

We first consider the Fashion-MNIST dataset (Xiao et al., 2017) by a two layer neural network. The first layer (FC1) is a 784 × 500 fully connected layer with a ReLU activation and the second layer (FC2) is a 500 × 10 fully connected layer with the softmax activation. We convert FC1 as a 8-th order tensor and FC2 as a 4-th order tensor for the tensor-train decomposition. For the Tucker decomposition, we convert FC1 as a 4-th order tensor and FC2 into a 3-th order tensor.

Next we consider the CIFAR-10 dataset. We build a 6-layer convolutional neural network (CNN) containing 4 convolution layers and 2 fully connected layers. Each convolution layer has a kernel size of 3 × 3 and padding of 1. The number of channels in each convolution layer is 128, 256, 256, 256, respectively. The size of the first fully connected layer (FC1) is 512. A batch normalization layer and a ReLU activation layer is placed after each convolution and fully-connected layer. A maxpooling layer with kernel size of 2 × 2 is placed after the second and the fourth convolution layer.

#### 4.2.2. Numerical Results

We use the ADAM method to minimize the negative posterior to get an initial point, then shrink the rank according to equation (22). In Fashion-MNIST dataset, the initialization takes 50 epochs, and in Cifar-10 dataset, it takes 150 epochs. We searched the hyperparameters in the grid defined by β among [0.1, 0.2, 0.5, 1, 2, 5, 10] and α among [1, 2, 5] and picked the one with the best trade-off between accuracy and compression ratio. Afterwards, we generate *T* = 450 SGHMC samples aftering discarding the first 50 samples, or *n* = 10 SVGD samples. We evaluate the accuracy of this model using two criterion: the predictive log likelihood (LL) and the prediction accuracy. The results for different benchmarks using different tensor formats are shown in Table 3. We compare the proposed Bayesian learning with the optimization method that maximize a posterior (MAP) directly. It is shown that our tensor learning framework outperforms MAP in almost every case in terms of both the accuracy and the log likelihood (LL). The improvement in log likelihood indicates that our model can predict the uncertainty better than the MAP method. Besides, our method achieves a compression ratio of up to 98.8× in Fashion-MNIST and 127× in CIFAR-10 in terms of the number of model parameters compared with the baseline network. One SGD initialization of our method on the CIFAR-10 problem takes approximately 3 h to run on an NVIDIA Titan V GPU with 12GB of memory. Either sampling process (SVGD or HMC) takes less than 10 min.

**Table 3:** Results of different networks on two datasets.

		**#Parameters**	**MAP**		**Proposed**	
**Dataset**	**Network**	**(compression ratio)**	**LL**	**Accuracy**	**LL**	**Accuracy**
Fashion-MNIST	NN	3.97 × 10^5^(1×)	–0.7118	88.91%	–0.6730	89.41%
	TT-NN	2.63 × 10^4^(15.1×)	–0.6687	87.07%	–0.6337	87.78%
	HMC-BF-TT-NN	4.02 × 10^3^(98.8×)	–0.3317	88.24%	–0.3254	88.64%
	SVGD-BF-TT-NN	2.8 × 10^4^(14.1×)	–0.3317	88.24%	–0.3261	88.57%
	Tucker-NN	2.57 × 10^5^(1.54×)	–1.1673	87.20%	–1.0984	87.53%
	HMC-BF-Tucker-NN	3.10 × 10^4^(12.8×)	–1.2948	87.18%	–0.4405	88.18%
	SVGD-BF-Tucker-NN	3.10 × 10^4^(12.8×)	–1.2948	87.18%	–0.4705	87.86%
CIFAR-10	CNN	9.91 × 10^6^(1×)	-0.5337	91.54%	–0.5370	91.53%
	TT-CNN	6.93 × 10^5^(14.3×)	–0.6077	89.00%	–0.5329	90.13%
	HMC-BF-TT-CNN	7.83 × 10^4^(127×)	–0.3936	86.68%	–0.3623	88.01%
	SVGD-BF-TT-NN	7.83 × 10^4^(127×)	–0.3936	86.68%	–0.3419	88.41%

*LL, predictive log likelihood (the larger the better); TT, tensor train decomposition; Tucker, Tucker decomposition; BF, Bayesian low rank prior*.

**Figure 1:**
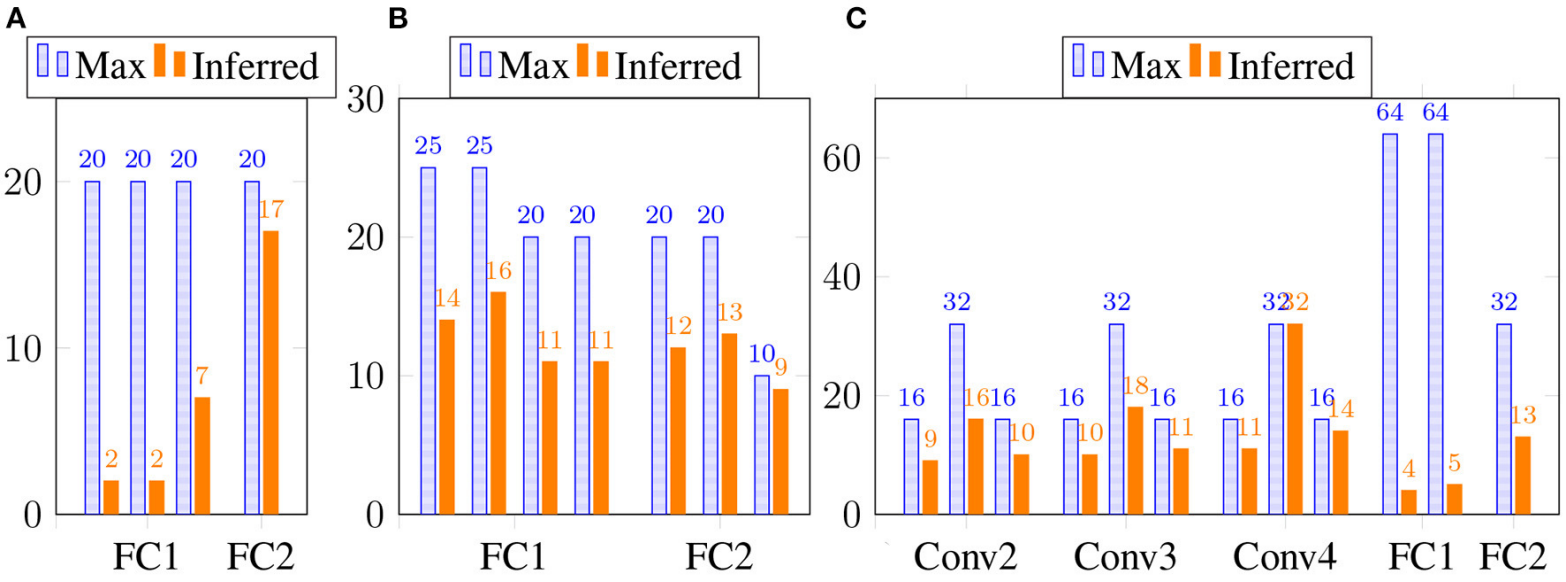
The inferred TT rank at different layers. **(A)** 2 TT-FC layers for Fashion-MNIST. **(B)** 2 Tucker-FC layers for Fashion-MNIST. **(C)** 4 TT-Conv and 2 TT-FC layers for CIFAR-10.

We also show the estimated tensor-train ranks of the estimated weight matrices and convolution filters in Figure 1. Clearly, our Bayesian tensor learning framework can perform model compression in the training process with automatic rank determination.

## 5. Related Work

There are many recent works on training a tensor learning model. There are a series of works targeting on tensor completion/factorization problems. Chu and Ghahramani (2009) proposed tensor completion algorithm using a maximum a posteriori (MAP) MAP estimation. Various attempts to evalute the uncertainly of completion using Bayesian method has been made in Xiong et al. (2010), Rai et al. (2014), Zhao et al. (2015a,c). On the other hand, some other works targets only on training a tensorized neural networks Jaderberg et al. (2014) demonstrated the first attempt to compress a CNN using tensor decomposition method. Tai et al. (2015) further improved that by training such a neural network from scratch and evaluate it on a large network. Kossaifi et al. (2019) proposed to compress a CNN by parametrizing the entire structure with a single, high-order tensor. In Kolbeinsson et al. (2021), tensor dropout technique was proposed. This technique can be applied to tensor factorizations and improve the robustness and generalization abilities while provide more computationally and memory efficient models. Bulat et al. (2021) further empirically demonstrated that tensor dropout method can improve the robustness to adversarial attacks. Hayashi et al. (2019) proposed a graphical notation to represent all kinds of decomposition used in tensorized CNN and experimentally compare the tradeoff between accuracy and efficiency. Our work proposes a generic, scalable and uncertainty-aware algorithm to solve a broad class of tensor learning problems, including but not limited to tensor factorization/completion, regression, and tensorized neural networks. Furthermore, these works either focused on post-training compression or fixed-rank training, while our work is the first to our knowledge that perform on-shot rank-adaptive training.

## 6. Conclusion

We have presented a generic Bayesian framework that is applicable to various tensor learning task described with different low-rank tensor representations. This framework is implemented with Hamiltonian Monte Carlo and Stein variational gradient descent. Among the wide range of applications in tensor learning tasks, we have specifically tested our methods by tensor completion with CP format and tensorized Bayesian neural networks with both tensor train and Tucker formats. In tensor completion, our method has shown better accuracy and capability of rank determination than the state-of-the-art mean-field approximation. In the Bayesian neural network, our method has demonstrated a significant compression ratio in the end-to-end training of tensorized neural networks, as well as better accuracy than the maximum-a-posterior training.

## Data Availability Statement

The raw data supporting the conclusions of this article will be made available by the authors, without undue reservation.

## Author Contributions

KZ and CH developed the low-rank Bayesian model under the guidance of ZZ. KZ coded the proposed HMC approach and CH coded the proposed SVGD approach. All authors contributed to the article and approved the submitted version.

## Funding

This work was supported by NSF CCF-1817037 and DOE ASCR grant no. DE-SC0021323.

## Conflict of Interest

The authors declare that the research was conducted in the absence of any commercial or financial relationships that could be construed as a potential conflict of interest.

## Publisher's Note

All claims expressed in this article are solely those of the authors and do not necessarily represent those of their affiliated organizations, or those of the publisher, the editors and the reviewers. Any product that may be evaluated in this article, or claim that may be made by its manufacturer, is not guaranteed or endorsed by the publisher.

## References

[B1] Acar E. Dunlavy D. M. Kolda T. G. Mørup M. (2011). Scalable tensor factorizations for incomplete data. Chemometrics Intell. Lab. Syst. 106, 41–56. 10.1016/j.chemolab.2010.08.004

[B2] Andrieu C. De Freitas N. Doucet A. Jordan M. I. (2003). An introduction to mcmc for machine learning. Mach. Learn. 50, 5–43. 10.1023/A:1020281327116

[B3] Austin W. Ballard G. Kolda T. G. (2016). Parallel tensor compression for large-scale scientific data, in Intl. Parallel and Distributed Processing Symp., (Chicago, IL: ACM), 912–922.

[B4] Bro R. (1997). Parafac. tutorial and applications. Chemometrics Intell. Lab. Syst. 38, 149–171. 10.1016/S0169-7439(97)00032-4

[B5] Bulat A. Kossaifi J. Bhattacharya S. Panagakis Y. Hospedales T. Tzimiropoulos G.. (2021). Defensive tensorization. arXiv preprint arXiv:2110.13859.

[B6] Candes E. J. Sing-Long C. A. Trzasko J. D. (2013). Unbiased risk estimates for singular value thresholding and spectral estimators. IEEE Trans. Signal Process. 61, 4643–4657. 10.1109/TSP.2013.227046427295638

[B7] Chen T. Fox E. Guestrin C. (2014). Stochastic gradient hamiltonian monte carlo, in International Conference on Machine Learning, (Beijing: ICML), 1683–1691.

[B8] Chu W. Ghahramani Z. (2009). Probabilistic models for incomplete multi-dimensional arrays, in Artificial Intelligence and Statistics, (Florida, FL: Clearwater Beach), 89–96.

[B9] Cichocki A. (2014). Era of big data processing: a new approach via tensor networks and tensor decompositions. arXiv preprint arXiv:1403.2048.

[B10] Ding N. Fang Y. Babbush R. Chen C. Skeel R. D. Neven H. (2014). Bayesian sampling using stochastic gradient thermostats, in Advances in Neural Information Processing Systems, (Vancouver, VN: MIT Press), 3203–3211.

[B11] Duane S. Kennedy A. D. Pendleton B. J. Roweth D. (1987). Hybrid monte carlo. Phys. Lett. B 195, 216–222. 10.1016/0370-2693(87)91197-X

[B12] Gilbert D. E. Wells M. T. (2019). Tuning free rank-sparse bayesian matrix and tensor completion with global-local priors. arXiv preprint arXiv:1905.11496.

[B13] Harshman R. A. (1970). Foundations of the PARAFAC procedure: Models and conditions for an “explanatory” multimodal factor analysis. UCLA Working Papers in Phonetics 16, 1–84. Available Online at: https://citeseerx.ist.psu.edu/viewdoc/download?doi=10.1.1.144.5652&rep=rep1&type=pdf.

[B14] Hawkins C. Zhang Z. (2018). Robust factorization and completion of streaming tensor data via variational bayesian inference. arXiv preprint arXiv:1809.01265.

[B15] Hawkins C. Zhang Z. (2021). Bayesian tensorized neural networks with automatic rank selection. Neurocomputing 453, 172–180. 10.1016/j.neucom.2021.04.117

[B16] Hayashi K. Yamaguchi T. Sugawara Y. Maeda S.-I. (2019). Exploring unexplored tensor network decompositions for convolutional neural networks, in Advances in Neural Information Processing Systems, Vol. 32 (Cambridge, MA:), 5552–5562.

[B17] Hillar C. J. Lim L.-H. (2013). Most tensor problems are np-hard. J. ACM 60, 45. 10.1145/2512329

[B18] Iserles A. (1986). Generalized leapfrog methods. IMA J. Numer. Anal. 6, 381–392. 10.1093/imanum/6.4.381

[B19] Jaderberg M. Vedaldi A. Zisserman A. (2014). Speeding up convolutional neural networks with low rank expansions. arXiv preprint arXiv:1405.3866.

[B20] Jain P. Oh S. (2014). Provable tensor factorization with missing data, in Advances in Neural Information Processing Systems, (Montreal, MO: MIT Press), 1431–1439.

[B21] Kingma D. P. Ba J. (2014). Adam: a method for stochastic optimization. arXiv preprint arXiv:1412.6980.

[B22] Kolbeinsson A. Kossaifi J. Panagakis Y. Bulat A. Anandkumar A. Tzoulaki I.. (2021). Tensor dropout for robust learning. IEEE J. Sel. Topics Signal Process. 15, 630–640. 10.1109/JSTSP.2021.306418227295638

[B23] Kolda T. G. Bader B. W. (2009). Tensor decompositions and applications. SIAM Rev. 51, 455–500. 10.1137/07070111X

[B24] Kossaifi J. Bulat A. Tzimiropoulos G. Pantic M. (2019). T-net: Parametrizing fully convolutional nets with a single high-order tensor. Proceedings of the IEEE/CVF Conference on Computer Vision and Pattern Recognition. Long Beach, CA, USA.

[B25] Liu Q. Wang D. (2016). Stein variational gradient descent: a general purpose bayesian inference algorithm, in Advances in Neural Information Processing Systems, Vol. 29 (Cambridge, MA: MIT Press), 2378–2386. PMC6923147 31857781

[B26] Neal R. M. (1992). Bayesian Training of Backpropagation Networks by the Hybrid Monte Carlo Method. Technical Report, Department of Computer Science, University of Toronto

[B27] Novikov A. Podoprikhin D. Osokin A. Vetrov D. P. (2015). Tensorizing neural networks, in Advances in Neural Information Processing Systems, (Cambridge, MA: MIT Press), 442–450.

[B28] Oseledets I. V. (2011). Tensor-train decomposition. SIAM J. Sci. Comput. 33, 2295–2317. 10.1137/090752286

[B29] Rai P. Wang Y. Guo S. Chen G. Dunson D. Carin L. (2014). Scalable bayesian low-rank decomposition of incomplete multiway tensors, in International Conference on Machine Learning, (Glasgow: ICML), 1800–1808.

[B30] Schmidt M. N. Mohamed S. (2009). Probabilistic non-negative tensor factorization using markov chain monte carlo, in European Signal Processing Conf., (Glasgow: ICML), 1918–1922.

[B31] Sidiropoulos N. D. De Lathauwer L. Fu X. Huang K. Papalexakis E. E. Faloutsos C. (2017). Tensor decomposition for signal processing and machine learning. IEEE Trans. Signal Process. 65, 3551–3582. 10.1109/TSP.2017.269052427295638

[B32] Şimşekli U. Koptagel H. Güldaş H. Cemgil A. T. Öztoprak F. Birbil Ş. İ. (2015). Parallel stochastic gradient markov chain monte carlo for matrix factorisation models. arXiv preprint arXiv:1506.01418.

[B33] Tai C. Xiao T. Zhang Y. Wang X. Weinan E. (2015). Convolutional neural networks with low-rank regularization. arXiv preprint arXiv:1511.06067.

[B34] Tucker L. R. (1966). Some mathematical notes on three-mode factor analysis. Psychometrika 31, 279–311. 10.1007/BF02289464 5221127

[B35] Xiao H. Rasul K. Vollgraf R. (2017). Fashion-mnist: a novel image dataset for benchmarking machine learning algorithms. arXiv preprint arXiv:1708.07747.

[B36] Xiong L. Chen X. Huang T.-K. Schneider J. Carbonell J. G. (2010). Temporal collaborative filtering with bayesian probabilistic tensor factorization, in Proceedings of the 2010 SIAM International Conference on Data Mining, (Beijing: SIAM), 211–222.

[B37] Zhang Z. Weng T.-W. Daniel L. (2016). Big-data tensor recovery for high-dimensional uncertainty quantification of process variations. IEEE Trans. Compon. Packag. Manufact. Technol. 7, 687–697. 10.1109/TCPMT.2016.262870327295638

[B38] Zhang Z. Yang X. Oseledets I. V. Karniadakis G. E. Daniel L. (2014). Enabling high-dimensional hierarchical uncertainty quantification by anova and tensor-train decomposition. IEEE Trans. Comput.-Aied Design Integr. Circuits Syst. 34, 63–76. 10.1109/TCAD.2014.236950527295638

[B39] Zhao Q. Zhang L. Cichocki A. (2015a). Bayesian cp factorization of incomplete tensors with automatic rank determination. IEEE Trans. Pattern Anal. Mach. Intell. 37, 1751–1763. 10.1109/TPAMI.2015.2392756 26353124

[B40] Zhao Q. Zhang L. Cichocki A. (2015b). Bayesian sparse tucker models for dimension reduction and tensor completion. arXiv preprint arXiv:1505.02343.

[B41] Zhao Q. Zhou G. Zhang L. Cichocki A. Amari S.-I. (2015c). Bayesian robust tensor factorization for incomplete multiway data. IEEE Trans. Neural Netw. Learn. Syst. 27, 736–748. 10.1109/TNNLS.2015.2423694 26068876

